# Mental Health Support Intervention for Ethiopian and Eritrean Youth: Protocol for Development and Pilot Testing of the Weyera Project

**DOI:** 10.2196/89155

**Published:** 2026-04-24

**Authors:** Tigest Fisseha Mekonnen, Tsedenia Tewodros, Feaven Gebrezgi, Rebbeca Tesfai, Janeria A Easley, Nioud Mulugeta Gebru, Antonio Newman Jr, Helen Fessahaye, Senait Kebede, Sophia Ahmed Hussen

**Affiliations:** 1 Hubert Department of Global Health, Emory University, 1518 Clifton Rd NE, Atlanta, GA, 30322, United States, 1 6785928836; 2 Department of Sociology, Temple University, Philadelphia, PA, United States; 3 Department of African American Studies, College of Art and Sciences, Emory University, Atlanta, GA, United States; 4 Department of Family and Preventive Medicine, School of Medicine, Emory University, Atlanta, GA, United States; 5 Emory Counseling and Psychological Services, Emory University, Atlanta, GA, United States; 6 Department of Medicine, Division of Infectious Diseases, Emory University, Atlanta, GA, United States

**Keywords:** Ethiopian or Eritrean youth, psychoeducation, coping skills, mental health education, resilience

## Abstract

**Background:**

Nearly 1 in 5 Black Americans are first- or second-generation immigrants; however, little research to date focuses on the experiences of these communities in a disaggregated and culturally specific way. Emerging adults (ages 18‐29) from Black immigrant backgrounds face multiple intersecting challenges to their mental health as they try to transition to adulthood and develop their identity, all while navigating intergenerational traumas from the immigration process and ongoing challenges faced as Black people in the United States. Our preliminary research has identified significant mental health disparities affecting Ethiopian and Eritrean emerging adults in Atlanta. Despite these threats to mental health and well-being, and the growing representation of Ethiopian, Eritrean, and other immigrant groups within the larger Black population, there are no evidence-based interventions that have been developed or tested specifically for Black immigrant emerging adults.

**Objective:**

This study aims to develop and pilot-test Weyera, a novel group-level intervention facilitated by trained peer facilitators aimed at enhancing resilience and improving mental health in this population. In phase 1, we will develop Weyera, a culturally responsive mental health support intervention for Ethiopian and Eritrean emerging adults. In phase 2, we will conduct a pilot trial of Weyera to evaluate feasibility, acceptability, and safety.

**Methods:**

In phase 1, we will use the intervention mapping approach and work with our established Youth Advisory Board (comprised of Ethiopian and Eritrean emerging adults) to refine an intervention outline and develop objectives and activities through a participatory, iterative process. In phase 2, we will pilot Weyera in a randomized waitlist-controlled trial, with participants randomized upon enrollment to an immediate intervention group or a delayed intervention (wait-list control) group. Participants will attend a 2-hour weekly group session for 8 weeks. Our evaluation will primarily focus on feasibility, acceptability, and safety, while also exploring potential intervention impacts on hypothesized effect modifiers (eg, resilience processes such as social support, affirming ethnic identity, and mental health service use) and mental health outcomes (eg, depression, anxiety, and trauma). We will assess our primary and secondary outcomes using mixed methods, including serial surveys as well as qualitative exit interviews, and we will also conduct process evaluations to monitor fidelity and adoption.

**Results:**

Phase 1, the development of Weyera activities, began in January 2025 and was completed in the summer of 2025. Phase 2, the implementation of the Weyera wait-list control trial, commenced in September 2025 and is projected to be finalized by April 2026. The final results are expected in summer 2026. As of March 2026, the study has enrolled 65 participants. This project was funded in September 2024.

**Conclusions:**

The trial is poised to demonstrate that the Weyera intervention is feasible, acceptable, and safe among Ethiopian and Eritrean immigrant youth. This work will add to the knowledge of using group-level interventions facilitated by trained peer facilitators to enhance resilience and improve mental health in this population.

## Introduction

Immigrants constitute a large and growing proportion of the US Black population. An estimated 10% of Black Americans are foreign-born, and another 8% are second-generation Americans. Within this group, the population of African immigrants is increasing rapidly, increasing by 246% between 2000 and 2019 [1]. Despite this rapidly growing number of immigrants, research on disaggregated African immigrants or their children and the health needs of specific African immigrant populations remains poorly understood. This study aims to fill this gap and provide significant insights into the mental health needs of Ethiopian and Eritrean youth [2,3]. Ethiopia and Eritrea, neighboring countries in Eastern Africa, constitute the second-largest area of origin for African immigrants in the United States [4,5]. These 2 neighboring countries share many cultural elements, as well as several instances of political violence, conflict, and natural disasters like severe droughts and famines. Ethiopian and Eritrean immigrants include refugees fleeing political violence, students, professionals, and/or individuals entering through diversity visa and family reunification initiatives [6,7]. A recent publication indicates that Ethiopians and Eritrean youth and communities in the United States often identify with this broader pan-ethnic group; thus, in this paper, we will examine Ethiopians and Eritreans together [8,9].

As immigrants of Ethiopian and Eritrean heritage in the United States navigate challenging and often traumatic circumstances, they are racialized as Black and subjected to both structural and interpersonal racism. As a result, Ethiopian and Eritrean immigrants and their children face significant challenges to their mental well-being. However, there is a paucity of research addressing mental health issues among Ethiopian and Eritrean (or any African) immigrants to date [10]. A cross-sectional study from Canada demonstrated that both premigration and postmigration stressors are linked to increased depression among Ethiopian immigrants and refugees [11]. Multivariate analysis in the same study found a significant association between adverse immigration-related experiences and depressive symptoms, which aligns with findings from our preliminary survey of Ethiopian and Eritrean immigrants in the United States [11].

Our preliminary work took place in Atlanta, which is home to one of the largest Ethiopian and Eritrean communities in the United States [4,5]. Anecdotal reports indicate a growing mental health crisis among emerging adults (18‐29 y old) in this community. Additionally, local leaders have shared reports of stress, depression, and even suicides; however, there is limited data that investigates the mental health situation in this population. Our 2022‐2023 study involved a 287-item online self-administered survey, completed by 200 Ethiopian and Eritrean emerging adults (ages 15‐25) in Atlanta [12]. The survey measured depressive and anxiety symptoms, sociodemographic characteristics, stressors, and resilience-related constructs. The findings indicated high rates of depression and anxiety among our sample, mainly consisting of 1.5 and second-generation college students, with 49.2% (98.4/200) of study participants reporting depressive symptoms above the clinically significant threshold, and 57.4% (114.8/200) meeting the criteria for mild, moderate, or severe anxiety. Stressors related to personal or parental immigration experiences, fear of failure, and discrimination were linked to higher levels of depression and anxiety, whereas food security and resilience were protective factors. Our findings also show that Ethiopian and Eritrean American youth report depression and anxiety linked to discriminatory experiences, most often citing race as the cause. Living at the nexus of Black and immigrant identities, youth may feel stress from both internalized high expectations, as those set by immigrant parents, and from limited opportunities due to the broader marginalization of Black Americans. These conflicting forces may together create unique challenges for Ethiopian and Eritrean immigrant youth [12,13].

Our exploratory qualitative analysis identified challenges faced by Ethiopian emerging adults as they manage their intersectional identity, cope with personal and/or intergenerational trauma from the immigration process, confront societal racism and discrimination, and encounter community norms that often discourage mental health care-seeking [13]. Our study found that Ethiopian adolescents and young adults are affected by multiple stressors that harm their mental health, with challenges amplified by individual, family, and community barriers to accessing professional care. Study participants report stressors such as intense academic pressure, cultural disconnects between parents and emerging adults due to differing acculturation or assimilation rates, and, in general, a deprioritization of mental health at home and within the larger Ethiopian community. Many of these challenges stem from the immigration experience, where parents often focus on immediate survival needs, for example, work, navigating financial difficulties, and achieving permanent residency, which leaves little room to address mental health issues and wellness. This context contributes to a high rate of undiagnosed mental health issues, particularly anxiety, depression, and suicide, among Ethiopian youth [13].

Despite these high levels of symptomatology, the growing population size, and the myriad social challenges they face, there are currently no published evidence-based mental health interventions designed to support Ethiopian and Eritrean emerging adults or any group of African immigrant youth in the United States. Community-based, peer-led mental health interventions have demonstrated acceptability, feasibility, and effectiveness among other immigrant groups [14-19]. Currently, there are no published interventions specifically targeting mental health issues for any African immigrant youth in the United States. However, research with other immigrant groups has shown that group mental health interventions led by peers or volunteers are acceptable and feasible and can improve self-rated anxiety and happiness levels [18], depressive symptoms [17], and psychosocial functioning while decreasing social isolation [16]. The group sessions within these interventions typically included psychoeducation and coping skills training; session durations ranged from 1.5 to 2 hours and were conducted weekly or biweekly over a period of 4 to 12 weeks. These programs were frequently facilitated by peer facilitators (PFs) who received training in mental health and group facilitation, thereby increasing the accessibility of these interventions. To address the needs identified in the community, we developed Weyera (Wellness: Ethiopian or Eritrean Youth Exploring Resilience and Awareness), a culturally responsive mental health support intervention for Ethiopian and Eritrean emerging adults, in collaboration with youth and community partners.

## Methods

### Study Objective

The overall objective of this study is to develop and pilot test Weyera to assess feasibility, acceptability, and safety before conducting a fully powered trial. We aim to accomplish this through two specific objectives: (1) to develop Weyera, a culturally responsive mental health support intervention for Ethiopian and Eritrean emerging adults, in partnership with youth and community stakeholders; and (2) to conduct a pilot trial of Weyera and evaluate its feasibility, acceptability, and safety among emerging adults in Atlanta.

### Ethical Considerations

The Institutional Review Board at Emory University has reviewed and approved all procedures outlined in this protocol (STUDY00008231 approved on August 25, 2024). The study is registered with ClinicalTrials.gov (NCT06960187). Our team works closely with a Licensed Master Social Worker, who is on-call during all sessions, to consult the PFs in case participants exhibit signs of a mental health crisis or distress. PFs are also trained in the correct referral methods in such situations. Informed consent will be obtained by the research team during the initial Zoom call before enrollment into the study. The team will explain the potential risks and adverse effects associated with participation, including potential discomfort or unpleasant experiences during group activities, as well as the possibility of confidentiality breaches. All participants are also provided with a list of affordable local mental health resources upon enrollment. Additionally, participants are encouraged to step away if they feel uncomfortable during a session. If any study participant reports feeling uncomfortable or unsafe during any session, the study team will work to address the issue as best as possible and offer the participant the option to withdraw from the study at any time. The study team will also track and report any adverse events to the Institutional Review Board. Participants will receive US $25 compensation for each session attended.

### Study Settings

This protocol is being carried out in Atlanta, home to one of the largest Ethiopian and Eritrean communities in the United States [4,5]. We work with 2 community-based organizations (CBOs) partners in the area, the Ethiopian Community Association of Atlanta and the Eritrean American Community Association of Georgia.

### Intervention Overview

**Figure 1.**
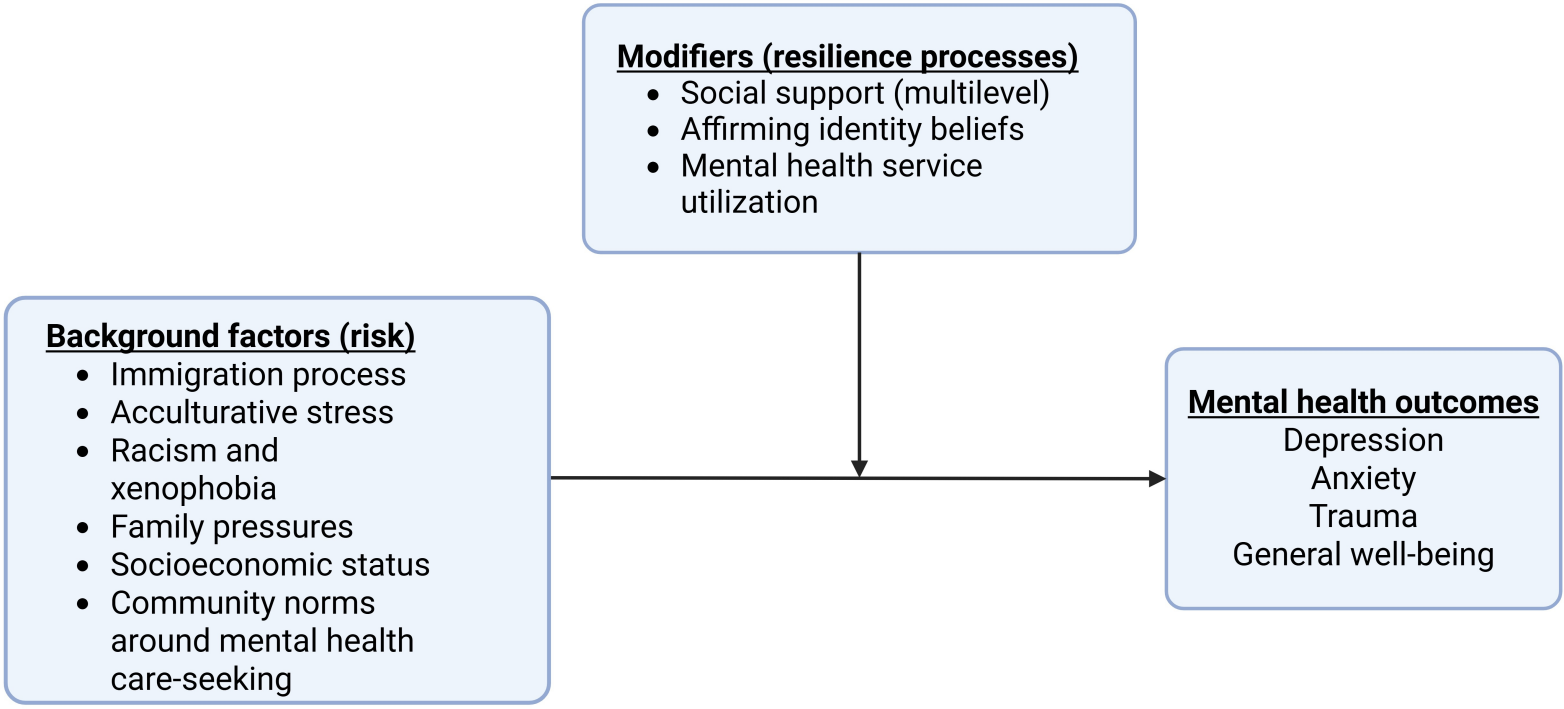
Conceptual model.

Our intervention is based on a conceptual model (Figure 1) grounded in Fergus and Zimmerman’s theorization of resilience as including assets (internal to the individual) and resources (external to the individual) [20]. Resilience is defined as the achievement of positive health outcomes despite risk [21], with resilience factors acting as effect modifiers on the expected risk-outcome relationship [20]. Our preliminary work identified resilience processes operating at multiple levels within Ethiopian and Eritrean communities, including strong family ties, affirming ethnic identity beliefs, and a culture of collective efficacy and support. Weyera aims to strengthen resilience assets and resources, such as social support, positive identity beliefs, and mental health service use, thereby reducing the adverse effects of background risk factors like immigration-related trauma, acculturative stress, racism, socioeconomic challenges, and harmful community norms on mental health outcomes such as depression, anxiety, and trauma among Ethiopian and Eritrean emerging adults. Of note, Weyera translates to “olive tree” (a symbol of peace) in Amharic, a language of Ethiopia.

### Participatory Approach

Our study is grounded in principles of community-based participatory research [22], to increase cultural relevance, community ownership, uptake, and sustainability [23]. To this end, in addition to partnering with the CBOs, we assembled a Youth Advisory Board (YAB) to cocreate, develop, or design the intervention. Our current YAB includes 13 members (n=5 men, n=8 women; n=5 Eritrean, n=8 Ethiopian). They were recruited through community outreach and social media advertisements. YAB duties include (1) advising on recruitment and retention; (2) reviewing surveys and interview guides; (3) finalizing design and testing pilot intervention sessions; (4) participatory data analysis for context; and (5) assisting with dissemination. YAB members are compensated at an hourly rate (US $15/h) for their involvement in this study. Our intervention development approach will be guided by the intervention mapping framework, an established, iterative process for systematically developing theory-based interventions, and builds on our team’s extensive work [19]. The investigative and analytic team consists of first- and second-generation Ethiopian American and African American faculty and students.

### Study Design

#### Overview

Phase 1 of this study, Weyera’s intervention development approach is guided by the intervention mapping framework [19], a proven, repeatable method for systematically creating interventions based on theory. In phase 2, we will conduct a wait-list control trial. Participants will be randomized upon enrollment to either an immediate intervention group or a delayed intervention (wait-list control) group, in which they will receive the intervention after a 2-month waiting period. Randomization will be done using the National Institutes of Health clinical trial randomization tool [24] to randomly generate a sequence. At enrollment, participants are assigned a study ID. Participant study ID will be entered into the online randomization software to assign participants into arm 1 or arm 2. A coin flip will then be used to designate one arm as the intervention and the other as the wait-list control group. Study allocation will be concealed from study participants and will only be limited to study staff. Participants are notified of their study group start date and all subsequent sessions. This study followed the CONSORT (Consolidated Standards of Reporting Trials) extension for pilot and feasibility trials, the TIDieR (Template for Intervention Description and Replication) checklist, and the SPIRIT (Standard Protocol Items: Recommendations for Interventional Trials) guidelines (Checklists 1-3).

#### Phase 1: Intervention Development

##### Development of the Logic Model

Major themes from our previous research are synthesized with the formative work, along with our theoretical and conceptual foundations, to create a logic model of the problem, outlining more specific influences on mental health challenges (the problem) among Ethiopian and Eritrean emerging adults. Behavioral and environmental conditions are delineated that require addressing mental health challenges and specifying the determinants of these conditions. We then create specific change objectives or concrete steps grounded in our preliminary research fin

dings. We combine objectives and determinants into matrices of change objectives, ultimately creating a logic model of change for the intervention. We created the logic model of the problem based on our formative work (past), outlined the change objective, and we will (future) work with the YAB to refine and finalize our logic model of change (draft version is in Textbox 1).

Textbox 1. **The logic model.**

Goal: To build resilience (assets and resources) through group sessions facilitating open dialogue, education, and skills practice.

- Inputs
  - Staff time
  - Peer facilitators (PFs) recruitment and training
  - REDCap (Research Electronic Data Capture) programming and maintenance
  - Youth Advisory Board input on session design
  - Formative mixed methods data
  - Community-based organizations partnership
- Activities
  - Develop interactive modules on psycho-education, coping skill development
  - Train PFs on modules, facilitation skills, trauma-informed care, and cognitive behavioral therapy
  - Run 8 weekly sessions with groups of 6‐10 Ethiopian and Eritrean youth
- Outputs
  - Culturally relevant facilitation guide
  - Six PFs trained in mental health and facilitation skills
  - Eight group mentorship sessions facilitated
  - Session materials and mental health resource guide provided
- Outcomes
  - Changes in knowledge
    - Increased awareness of symptoms, treatments of common mental health conditions & resources
  - Changes in perception
    - Increased social support from peers or PFs
    - Decreased stigma toward mental health
    - Increased affirming ethnic identity beliefs
    - Increased perceived personal resilience
    - Improved mental health seeking attitudes
  - Changes in behavior
    - Increased use of evidence-supported practices for mental wellness or coping
    - Increased use of mental health services
- Long-term impact
  - Improvements in mental health outcomes (depression, anxiety, and posttraumatic stress disorder)

Assumptions (based on pilot data and literature): (1) Community-based participatory research and intervention mapping as guides for iterative development; (2) resilience factors act as effect modifiers on the expected risk-outcome relationship for Ethiopian and Eritrean emerging adults; and (3) PF-led interventions effective for immigrants.

##### Program Design and Production

Through an iterative design process with our YAB, Weyera has been developed as an 8-week program consisting of weekly 2-hour group sessions with 6‐10 participants, led by 2 trained lay facilitators. Based on initial findings and input from the YAB, key modules for the program include (Table 1) the following: (1) introduction to mental health; (2) stress and family pressure; (3) self-exploration and critical reflection; (4) intersectional identities; (5) family relationships; (6) discussing mental health within the Ethiopian and Eritrean community; (7) trauma and abuse; and (8) resilience. Each session will cover the weekly topic and introduce a research-backed coping strategy, such as mindfulness or positive refocusing, for participants to practice during the week.

We pretested intervention materials and modules by presenting them to the YAB, followed by collaborative meetings to gather feedback on content, language, and any necessary redesign. YAB members and the study team documented concerns and suggestions during these sessions, and the team reviewed, revised, and finalized the manual.

**Table 1.** Intervention modules and activities described.

Modules	Description	Activities	
1. Getting acquainted	Getting to know each other Defining mental health Defining mental health in the Ethiopian and Eritrean community	My group’s norms and guidelines Words of affirmation	
2. Self-exploration	Discuss the benefit of understanding ourselves and journal about life lessons	Values mood board Journaling activity	
3. Pressures and fears	Discuss the pressure to succeed, the fear of failure How stress affects the body Discuss cognitive distortions and practice reframing our thoughts	Distorted thinking patterns and cognitive reframing Bucket list	
4. Family relationships	Discuss relationships with family, how those relationships affect mental health Tips on communicating with family members	Show and tell: family edition Journaling activity	
5. Navigating intersectional identities	Discuss how we navigate our different identities Discuss how race and ethnicity are connected to mental wellness	Mask activity: the different masks we wear	
6. Mental health issues in our community	Explore the different mental health issues that are common in the communities Talk about therapy and medication as treatments for mental health issues How to access psychotherapy when it’s needed	How to book a consultation with a therapist Practice questions to ask when establishing care	
7. Trauma	Discuss trauma, adverse childhood experiences How to make the communities more trauma-informed	Grounding activity (what to do in the moment) Safety plan (what to do in the future)	
8. Resilience and program reflection	Talk about resilience—how all these aspects come together to help build our ability to cope and emerge from difficult experiences Reflect on the last 8 weeks and then we say goodbye!	Personalized affirmation for another participant in the group	

##### Peer Facilitators Training and Roles

We recruited and trained 8‐10 Ethiopian and Eritrean emerging adults as PFs, who will deliver the intervention content during the pilot trial. Recruited PFs are Ethiopian and Eritrean emerging adults who are connected to the local community. Most of the YAB members volunteered to serve as PFs for the intervention; we also identified 2 additional emerging adults to serve as PFs through social media advertisements and peer networks. Training domains included the Weyera intervention manual, group facilitation techniques, psychoeducation (including how to navigate mental health crises), and other modalities used in immigrant-focused PFs intervention research [14-18,25-27]. Trainings also included practice sessions in which PFs delivered Weyera modules to each other via Zoom while study team members observed, provided feedback, and noted differences to be resolved before the trial. We created 4 PF teams (with 2‐3 PFs in each team), enabling us to run 4 groups simultaneously. During the trial, PFs have weekly planning meetings within their teams prior to facilitating the session for that week and larger group meetings to debrief and exchange ideas after each weekly session. We will continue to meet weekly with PFs during the trial to share experiences and troubleshoot any concerns related to intervention fidelity.

### Phase 2: Pilot Trial

#### Overview

We will conduct a wait-list control trial, where participants are randomized upon enrollment to either an immediate intervention or a delayed intervention (wait-list control) condition, in which they receive the intervention after a 2-month waiting period. We chose this design to protect the ethical treatment of participants by ensuring that everyone will have access to a potentially impactful intervention after a short waiting period [28,29].

#### Participant Experience

For phase 2, we will recruit a total of 60 participants. Inclusion criteria include (1) Ethiopian and/or Eritrean origin; (2) age 18‐29 years (consistent with Arnett’s conceptualization of emerging adulthood [30], and community priorities); (3) residence in the Atlanta area; and (4) ability to read and comprehend English. Eligibility will be assessed via a brief screener survey, which will be self-administered remotely (eg, if responding to a social media advertisement) or on a tablet by study staff (if recruited in-person). Study participants will be recruited using multiple approaches, including CBO and student organizations, YAB referrals, social media recruitment, and venue-based recruitment at community cultural events. Study team members will contact those who express interest for an initial enrollment meeting. During this initial Zoom meeting, a study team member will conduct a one-on-one informed consent discussion before enrolling participants. During the consent procedure, the research team will detail the intervention components and address any inquiries raised by the participant. The team will also outline the potential risks and harms associated with participation in this study, including possible unpleasant or uncomfortable experiences during group activities with other Ethiopian and Eritrean youth, as well as the risk of a breach of confidentiality. Measures will be implemented to ensure that personal information remains confidential and is not accessible to individuals outside the research team; this includes using passwords and encryption to secure digital study files. Physical documents will be stored in a locked file cabinet accessible solely to the research team. However, if a study participant discloses personal information during the group session, there is a possibility that other participants will share it outside the session. The consent process will be completed electronically using RedCap (Research Electronic Data Capture) [31]. After the initial study overview walkthrough, the study team will share the consent form with the participant via Zoom chat or email, depending on the participant’s preference. The participant will have the opportunity to review the consent documents and ask any questions before signing the electronic consent form provided through RedCap. A signed copy of the consent form will be sent to participants for their records. Participants will also complete a baseline survey (measuring risk factors, resilience processes, and mental health outcomes) and will then be randomly assigned to either the immediate or delayed intervention group. We will recruit and enroll continually until we accrue groups of 16 individuals. Within these groups, we will randomly assign half (n=8 people) to the immediate intervention and half (n=8 people) to the delayed intervention condition. This process will be repeated until we reach our overall recruitment goals.

Participants will attend a 2-hour group session once per week for 8 weeks. The first and final sessions are designed to be delivered in person, while the remainder is to be delivered via Zoom videoconference. A total of 2 or 3 trained PFs will lead sessions. The structure, duration, and length of the program were designed by the study team together with the YAB and also reflect previous group interventions developed for other immigrant groups [15,16,18]. Sessions will include psychoeducation, engaging, interactive activities (eg, case-scenario discussions and trivia games), and practice of coping skills (Table 1). Participants will complete a session evaluation form each week and receive US $25 payment on an electronic gift card. All participants will be provided with a mental health resource guide featuring affordable services verified and compiled for Ethiopian and Eritrean youth in Atlanta. After completing the program, participants will receive links to follow-up surveys at 2, 4, and 6 months, and a subset will take part in qualitative interviews.

### Sample Size and Power

The goals of this study are to assess feasibility, acceptability, and safety. The proposed sample size of 60 participants was not derived based on estimating effect size estimates (which are often unreliable from small pilot studies [32-41]), but to provide a sufficiently large sample to obtain reasonable descriptive data and to pilot test our data collection procedures, intervention manual, and recruitment and retention methods, in anticipation of a future fully powered efficacy trial. The qualitative sample size is based on Kvale’s estimate that 15±5 individuals are typically sufficient to reach thematic saturation [42].

### Data Collection

We will use REDCap [31] to create and allow self-administration of surveys measuring background factors, resilience processes, and mental health outcomes at baseline 2-, 4-, and 6-month time points (Table 2). Surveys will primarily be completed remotely but can be done in person if a participant prefers. We estimate that completing each survey will take approximately 30 minutes. We will use best practices for web-based data collection to detect suspicious response patterns [43]. Upon completing the survey, respondents will receive US $25. Short postsession participant and facilitator surveys will capture satisfaction and challenges with each session. In addition to the survey measures outlined in Table 2, the study team will conduct a fidelity check at each session to ensure that each session closely follows the intervention design. We will use a participant attendance and engagement tracker to monitor participants’ engagement throughout each session closely.

**Table 2.** Survey measures.

Construct	Measure	Description	Number of items and examples	Response format
Relationships and roles	Multidimensional scale of perceived social support	Questionnaire measuring support system	12 items; eg, “There is a special person who is around when I am in need. Or my family really tries to help me.”	7-point Likert scale
Cultural and community factors	Phinney’s multigroup ethnic identity measure	Questionnaire measuring ethnic identity feeling or reaction	12 items; eg, “I have a clear sense of my ethnic background and what it means for me. Or I am active in organizations or social groups that include mostly members of my own ethnic group.”	4-point Likert scale
Subjective psychological well-being	General well-being	Questionnaire measuring subjective psychological well-being and distress	24 items; eg, “In the past month; how have you been feeling in general? Or, during the past month, have you been bothered by nervousness or your nerves?”	Likert scale and 6-point and 10-point scales
Knowledge, beliefs, and attitudes	Mental health knowledge, beliefs, attitudes, and coping strategies (MHLqSVa^a^)	Questionnaire measuring mental health knowledge, beliefs, and attitudes	16 items; eg, “Mental disorders do not affect people’s behavior. Or mental illness is a sign of personal weakness.”	5-point Likert scale
Depression symptoms	Patient health questionnaire-9	Screen for depression, measure its severity, and monitor treatment responses	9 items; eg, “Little interest or pleasure in doing things or feeling down, depressed, or hopeless.”	4-point scale
Anxiety symptoms	Generalized anxiety disorder-7	Questionnaire used to assess the severity of generalized anxiety disorder	7 items; eg, “Worrying too much about different things or feeling nervous, anxious, or on edge.”	4-point scale
Resilience	Brief resilience Scale	Questionnaire measures an individual’s ability to recover from stress	6 items; eg, “I have a hard time making it through stressful events or I usually come through difficult times with little trouble.”	5-point Likert scale
Trauma experiences	Posttraumatic stress disorder checklist (PCL)	Questionnaire used to screen and monitor PTSD symptoms	20 items; eg, ”Avoiding memories, thoughts, or feelings related to the stressful experience? Or trouble remembering important parts of the stressful experience?”	5-point scale
Stigma	Personal and perceived stigma (unvalidated questionnaire)	Questionnaire used to assess how others perceive people with mental health issues	15 items; eg, “Most people believe that a person who has received mental health treatment is as intelligent as the average person or most people feel that receiving mental health treatment is a sign of personal failure.”	6-point Likert scale
Stigma	Self-stigma of seeking help scale-3 (ultra-brief)	Questionnaire assessing reaction to a situation	3 items; eg, “It would make me feel inferior to ask a therapist for help. Or if I went to a therapist, I would be less satisfied with myself.”	5-point Likert scale
Coping strategies	Mental health behaviors (unvalidated questionnaire)	Questionnaire assessing coping strategies practiced in sessions	12 item; eg, “In the past 2 month how often have you used journaling to deal with stress or mental health distress?”	5-point Likert scale
Alcohol and drug use	NIAAA^b^ and DAST-10^c^	Self-reported use of consumption of alcohol and other substances	14 items; eg, “During the last month, on how many days did you have any kind of drink containing alcohol? By a drink we mean half an ounce of absolute alcohol (eg, a 12 ounce can or glass of beer or cooler, a 5-ounce glass of wine, or a drink containing 1 shot of liquor.)”	Yes or no
Self-efficacy	The general self-efficacy Scale, adapted	Questionnaire assessing confidence in ability to solve problems	10 items; eg, “I can always manage to solve difficult problems if I try hard enough.”	4-point Likert scale

a MHLqSVa: Mental Health Literacy questionnaire—short version for adults.

b NIAAA: National Institute on Alcohol Abuse and Alcoholism.

c DAST-10: Drug Abuse Screening Test.

### Qualitative Exit Interviews

To qualitatively evaluate feasibility and acceptability, we will conduct brief exit interviews with a subset of study participants (n=20), purposively sampled to represent different ethnicities, religions, genders, and immigrant backgrounds. Additionally, we will conduct interviews with each PF at the end of the study. These interviews will be conducted by trained staff via Zoom.

### Statistical Analysis

We will conduct descriptive analysis to assess the acceptability, feasibility, and safety of Weyera. To evaluate the acceptability of the intervention, descriptive quantitative analyses of the intervention data will focus on tabulating and summarizing the results of the postsession satisfaction survey measures. To determine feasibility, we will monitor recruitment rates, effort required (eg, staff hours), number of screenings, and the proportion of eligible participants who agree to enroll. We will summarize rescheduled, canceled, and missed sessions to help estimate staffing needs and retention protocols for a future full trial. Additionally, we will compare the characteristics of participants lost to follow-up with those who remain to identify any systematic patterns that may affect the results. Safety will be assessed by tabulating and describing any adverse events during study participation.

Additionally, we will measure mental health outcomes, hypothesized moderators, and background (risk) factors to evaluate the feasibility of data collection (eg, time to complete surveys), as depicted in Table 1. To analyze changes over time, we will plot means and proportions from baseline to follow-ups. Exploratory hypotheses will be evaluated using linear mixed models, estimated via maximum likelihood, and fitted to provide all necessary information for analyses typical of a full randomized controlled trial [44]. We will also assess the feasibility of interaction analyses (for effect modification). As of March 2026, the study has enrolled 65 participants (33 in the intervention group, 32 in the control group) who have completed the baseline survey. We have not started data analysis.

### Qualitative Analysis

Qualitative analyses will be guided by a thematic analysis and use a team approach to enhance rigor and reproducibility [45]. Analytic steps will include the following: (1) after each interview, interviewers will document field notes including emerging topics for further exploration; (2) verbatim transcripts will be entered into MAXQDA qualitative software (VERBI Software GmbH); (3) we will develop a preliminary codebook to include predetermined deductive codes related to the domains of interest, and inductive codes that emerge; (4) analysts will code a subset of transcripts and compare coding, with differences discussed in team meetings until consensus is reached; similarities and differences across transcripts will be examined, revising codes accordingly. We will cease developing new codes when no new themes are seen; (5) analysts will proceed to individually code full transcripts and meet weekly to troubleshoot concerns and; (6) properties and dimensions of salient themes will be summarized and interpreted in detailed analytic memos.

## Results

Phase 1 (development) of Weyera research activities began in January 2025 and was completed in June 2025. The data collection for the phase 2 pilot implementation trial began in September 2025 and is expected to be completed in April 2026. Final results are anticipated in summer 2026.

## Discussion

### Expected Findings

The trial is well positioned to make a significant contribution by demonstrating that the Weyera intervention is feasible, acceptable, and safe among Ethiopian and Eritrean immigrant youth. There are currently no published studies of interventions that specifically target the mental health needs of Ethiopian and Eritrean youth in the United States. Given this research gap, this trial will provide evidence on whether a community-based, peer-led mental health intervention can begin to address the mental health needs of this community. Studies involving different immigrant groups indicate that peer-led mental health interventions are both acceptable and feasible, and these programs can improve self-rated anxiety and happiness levels, depressive symptoms [17], and psychosocial functioning while decreasing social isolation [16]. This trial builds on this evidence to specifically develop an intervention for and with Ethiopian and Eritrean immigrant youth.

### Limitation and Future Directions

This randomized wait-list control trial is designed to minimize bias. Beyond the study team, participants remain unaware of their specific group assignment; they are only notified of the start dates for their respective group sessions. Sessions for both the immediate and wait-list groups are designed and implemented identically. Nevertheless, this methodology does not entirely eliminate potential selection bias, given the inherent challenges associated with this study design, in which the wait-list group may be prone to higher dropout rates due to the interval between enrollment and the start of the intervention. Furthermore, varying expectations among participants may affect their motivation to fully engage with the intervention following the waiting period. In addition, the presurvey and postsurvey measures are self-reported by study participants, which can introduce biases due to reduced accuracy and potential social desirability bias.

Upon the conclusion of this trial, in collaboration with the CBOs, the team will conduct webinars to present our qualitative and quantitative findings. In addition, further dissemination will include scientific and academic journal publication and conference presentations. Building upon the findings of this research, the subsequent objective of the project is to undertake a statistically powered trial designed to evaluate the efficacy of the intervention in addressing the mental health needs of Ethiopian and Eritrean immigrant youth.

## Supplementary material

10.2196/89155 Checklist 1 Template for intervention description and replication checklist.

10.2196/89155 Checklist 2 SPIRIT 2025 checklist.

10.2196/89155 Checklist 3 CONSORT extension pilot and feasibility trials checklist.

10.2196/89155 Peer Review Report 1 Peer review report by the Health Promotion in Communities Study Section, Social and Community Influences on Health Integrated Review Group - National Institute on Minority Health and Health Disparities (National Institutes of Health, United States).

## Abbreviations

CBO: community-based organization
CONSORT: Consolidated Standards of Reporting Trials
PF: peer facilitator
REDCap: Research Electronic Data Capture
SPIRIT: Standard Protocol Items: Recommendations for Interventional Trials
YAB: Youth Advisory Board
