# Supplementary material for: Mental Health Support Intervention for Ethiopian and Eritrean Youth: Protocol for Development and Pilot Testing of the Weyera Project
Source: JMIR Res Protoc. 2026 Apr 24;15:e89155. doi: 10.2196/89155 (PMC13108575; doi:10.2196/89155)
Supplement: Peer Review Report 1 [file resprot-v15-e89155-s004.pdf]

1R21MD019304-01A1 Hussen, Sophia

RESUME AND SUMMARY OF DISCUSSION: This resubmitted application proposes to develop and pilot test a novel group-level intervention facilitated by trained community health workers to enhance resilience and improve mental health among Ethiopian and Eritrean (Habesha immigrants) emerging adults in Atlanta, Georgia. The panel agreed that the project's rigor of prior research is strong and if successful, it would help advance understanding of evidence-based mental health interventions adapted for Black immigrants, a population whose experiences are often hidden in aggregate samples of Black/African Americans. The panel found the application highly innovative as it would be the first to culturally adapt a mental health intervention for Habesha immigrants. The investigative team is also highly qualified and has relevant expertise to successfully achieve study aims. The research environment is also excellent with the necessary resources to support the project. The panel noted several major strengths with the project's rigorous approach. It builds on prior cross-sectional surveys conducted by the investigative team and uses well-established measures. The project utilizes an asset-based approach and is responsive to the study community's concerns. Relatedly, the panel commended the project for its engagement of the study community in the development and conduct of the intervention. The panel agreed that this resubmission was highly responsive to prior critiques and provides additional details on intervention mapping expertise, the rigor of prior research, and explanation of study measures. The panel also noted some minor weaknesses with the approach including, concern that the sample size of 60 may be inadequate to examine moderators and lack of justification for use of weekly assessments. However, these did not detract from the high enthusiasm the panel had for this application. Overall, this project is likely to have a high impact on informing mental health interventions among Black immigrant groups.

DESCRIPTION (provided by applicant): Nearly one in five Black Americans are first- or second-generation immigrants; however, little research to date focuses on the experiences of these communities in a disaggregated and/or culturally-specific fashion. Emerging adults (ages 18-29) from Black immigrant backgrounds face multiple intersecting challenges to their mental health, as they attempt to transition to adulthood and develop their identity, all while navigating intergenerational traumas from the immigration process as well as ongoing racism and discrimination faced as Black people in the United States. Our preliminary research has identified significant mental health disparities impacting Ethiopian and Eritrean (collectively referred to as habesha) emerging adults in Atlanta. Despite these threats to mental health and well-being, and the growing representation of habesha and other immigrant groups within the larger Black population, there are no evidence-based interventions that have been developed or tested specifically for Black immigrant emerging adults. Our long-term goal is to improve mental health among habesha emerging adults, as a first step towards ameliorating disparities impacting Black immigrants more broadly. The objective of this R21 application is to develop and pilot test Weyera, a novel group-level intervention facilitated by trained community health workers, with a goal to enhance resilience and improve mental health in this population. This study will pursue two specific aims: (1) To develop Weyera, a culturally- responsive mental health support intervention for habesha emerging adults; and (2) To conduct a pilot trial of Weyera and evaluate its feasibility, acceptability, and safety. For the first aim, we will use the Intervention Mapping approach and work with our established Youth Advisory Board (comprised of habesha emerging adults) to refine an intervention outline and develop objectives and activities through a participatory, iterative process. In the second aim, we will pilot Weyera in a randomized waitlist control trial. Our evaluation will primarily focus on feasibility, and acceptability and safety, while also exploring potential intervention impacts on hypothesized effect modifiers (resilience processes: i.e., social support, affirming ethnic identity, mental health service utilization) and mental health outcomes (depression, anxiety and trauma). We will assess our primary and secondary outcomes using mixed methods including serial surveys as well as qualitative exit interviews, and we will also conduct process evaluations to monitor fidelity and adoption.

Results from this study will provide the foundation for a larger efficacy trial of Weyera in the future. The proposed research is highly significant because of its potential to begin to address the critical gap of knowledge and intervention focused on habeshas or any Black immigrant group. Attention to understudied nuances including ethnicity, nativity and immigration status within the Black population is critical to achieving the NIH's overall goals of addressing the impacts of racism and discrimination on health.

PUBLIC HEALTH RELEVANCE: The proposed project seeks to develop and pilot test a mental health support intervention (Weyera) to build resilience among Ethiopian and Eritrean emerging adults. This project is relevant to public health because Black immigrant-origin groups, including Ethiopian and Eritrean emerging adults, are a large and growing population who encounter significant mental health challenges that remain unaddressed.

CRITIQUE 1

Significance: 2
Investigator(s): 1
Innovation: 2
Approach: 2
Environment: 1

Overall Impact: This R21 resubmission is very responsive to prior reviews. The investigators propose to develop and pilot test a resilience-based intervention for first and second generation Habesha emerging adults. The investigators will evaluate feasibility, acceptability, and safety of the intervention with 60 participants randomized to either intervention or delayed intervention. They will explore effects on mental health and whether resilience resources are moderators or effect modifiers. The proposal is expected to have high impact. This is a highly significant and innovative proposal by a strong team of investigators. The approach is rigorous and reproducible and address the weaknesses of prior research.

1. Significance:

Strengths

- No studies have developed mental health programs or policy efforts for habesha youth.
- The proposal is supported by strong preliminary studies demonstrating high prevalence of mental health symptoms among the population, that resilience is protective and that there is a critical need and desire for interventions.
- Prior studies support the feasibility of peer-led mental health interventions for immigrant groups.

Weaknesses

- None noted by reviewer.

2. Investigator(s):

Strengths

- Strong team of investigators with relevant expertise to complete the proposed study.

Weaknesses

- None noted by reviewer.

3. Innovation:

Strengths

- The population of first and second generation habesha emerging adults is innovative.
- Focus on asset-based intervention approach is innovative.
- Community partnerships focused on immigrant groups is underutilized, and innovative given the focus on mental health interventions.

Weaknesses

- None noted by reviewer.

4. Approach:

Strengths

- The proposed approach is rigorous and reproducible and will address weaknesses of prior research because it will test the intervention with an understudied population, focus on an asset-based approach, be conducted in collaboration with the target community, build on prior work research, and the majority of the measures are well-established in the field
- Proposed examples of the intervention, logic model and potential change objectives support the proposed intervention content.
- Sex as a biological variable is considered.

Weaknesses

- Approach to fidelity monitoring could be strengthened (minor).

5. Environment:

Strengths

- Team has relevant facilities and resources to complete the proposed work.

Weaknesses

- None noted by reviewer.

Study Timeline:

Strengths

- The timeline is well-detailed and supports feasibility of completing proposed study aims.

Weaknesses

- None noted by reviewer.

Protections for Human Subjects:

Acceptable Risks and/or Adequate Protections

- No concerns

Data and Safety Monitoring Plan (Applicable for Clinical Trials Only):
Acceptable
- No concerns

Inclusion Plans:
- Sex/Gender: Distribution justified scientifically
- Race/Ethnicity: Distribution justified scientifically
- Inclusion/Exclusion Based on Age: Distribution justified scientifically
- No concerns

Vertebrate Animals:
Not Applicable (No Vertebrate Animals)

Biohazards:
Not Applicable (No Biohazards)

Resubmission:
- The application is highly responsive to reviews.

Resource Sharing Plans:
Not Applicable (No Relevant Resources)

Budget and Period of Support:
Recommend as Requested

CRITIQUE 2

Significance: 2
Investigator(s): 2
Innovation: 2
Approach: 2
Environment: 1

Overall Impact: The application is a resubmission study seeks to develop and test a mental health intervention among Black immigrants. The investigators propose to develop the mental health intervention with the community's input and Aim 2 will conduct a randomized to immediate or waitlist pilot trial to test the feasibility, acceptability, and safety among sixty participants. Significance is that the proposal builds on previous research that supports the scientific premise and is supported by prior rigorous research. The intervention is innovative in that it the first to explore mental health interventions among Black immigrant young adults. The approach addresses gaps in previous research and is well described. The intervention development is guided by the community with strong qualitative methods to

include intervention mapping and sex as a biological variable is considered. The investigative team have the requisite backgrounds and experience to complete the aims and the environment at Emory and the combined community collaborators have the requisite resources, community support and access to the priority population. The investigators are very responsive to previous critiques and improved the application with additional details regarding intervention mapping expertise, rigor of previous research, fidelity, and explanation of measures. Overall, the application is strong and well described, if successful it will fill a critical gap in the research literature and will have high impact on the field.

1. Significance:

Strengths

- Major: There is a growing Black immigrant population who experience mental health challenges; however, their unique experiences are often hidden in aggregated samples as Black/African American.
- Moderate: The proposal addresses a major barrier regarding evidenced based mental health interventions adapted for Black immigrants, especially for young adults.
- Moderate: The scientific premise is derived from research drawn from other immigrant groups, community input, youth advisory group and preliminary data.

Weaknesses

- None noted by reviewer.

2. Investigator(s):

Strengths

- Major: The team has the expertise to conduct the trial; community based participatory mental health research among young adults; qualitative methods (PI); Easley (statistician); (Tesfai) expert on Black immigrant experiences; Community engagement with Ethiopian and Eritrean populations (Kebede); Hussen-intervention mapping framework.

Weaknesses

- Minor: The statistician has experiences predominately with economic outcomes.

3. Innovation:

Strengths

- Major: The proposed study is innovative in that it is the first to develop a culturally adapted mental health intervention among this subpopulation.

Weaknesses

- None noted by reviewer.

4. Approach:

Strengths

- Major: The intervention is informed by cross-sectional survey collected by the investigators indicates significant mental health disparities and that high resilience was associated with lower

mental health symptoms and the strong qualitative data indicates the mental health challenges of the population; this preliminary work informs the intervention components. Training the youth as CHW may increase acceptability.

- Major: Conceptual model and Social Cognitive Theory are included that shows the major intervention components that will be included.
- Minor: Sex as a biological variable was considered.
- Major: The project is responsive to community concerns, leverages on the strengths of the surrounding area (Atlanta) one of the largest Habesha communities.
- Major: The proposed project addresses a major gap in the research literature.
- Major: Attention to fidelity is well thought out.
- Major: The qualitative analysis and intervention mapping is well described.
- Moderate: Although the intervention is under development, the rationale for the conceptualization of length, topics, etc. are informed by the community.

Weaknesses

- Moderate: The sample size of 60 is insufficient to explore moderators.
- Minor: Although the Logic Model is included due to input from previous reviews, the outcomes are future focused and not well aligned with the expected outcomes of the proposed project.

5. Environment:

Strengths

- Major: Emory and Temple and the community partners have the requisite resources.
- Major: The youth advisory board is well established.

Weaknesses

- None noted by reviewer.

Study Timeline:

Strengths

- The timeline includes major activities and is feasible.

Weaknesses

- None noted by reviewer.

Protections for Human Subjects:

Acceptable Risks and/or Adequate Protections

Data and Safety Monitoring Plan (Applicable for Clinical Trials Only):

Acceptable

Inclusion Plans:

- Sex/Gender: Distribution justified scientifically

- Race/Ethnicity: Distribution justified scientifically
- Inclusion/Exclusion Based on Age: Distribution justified scientifically

Vertebrate Animals:

Not Applicable (No Vertebrate Animals)

Biohazards:

Not Applicable (No Biohazards)

Resubmission:

- The investigators are very responsive to previous critiques and improved the application with additional details regarding intervention mapping expertise, rigor of previous research, fidelity, and explanation of measures.

Budget and Period of Support:

Recommend as Requested

CRITIQUE 3

Significance: 4
Investigator(s): 1
Innovation: 2
Approach: 2
Environment: 1

Overall Impact: The application proposes to develop and pilot test a mental health support intervention for Ethiopian and Eritrean emerging adults. Due to the substantial population, the documented poor mental health in this community based on preliminary data, and NIH/NIMHD priorities to ameliorate disparities impacting immigrant populations, the proposed research has public health significance. If this moderately innovative intervention is effective, it could help fill a critical gap in evidence-based interventions that improve mental health among African immigrant emerging adults. However, it is not clear which existing evidence-based interventions it may be based on and whether a new intervention is needed (versus adapt an existing one). The investigative team has expertise in many areas needed to complete the research and includes community partners. The approach has many strengths; however, it is not clear that weekly assessments are necessary, and they may distort attendance/engagement estimates (given the incentives). The overall potential impact of this project is considered moderate-high.

1. Significance:

Strengths

- Moderate: The immigrant communities for which this intervention is proposed, constitute the second largest area of origin for African immigrants in the US.

- Moderate: This population of immigrants have high rates of depression, anxiety and trauma, and ameliorating disparities in immigrant populations is a priority. However, few habesha youth utilize mental health services.
- Moderate: There are specific challenges in this population, including mental health stigma, pressure to help parents navigate the US, and intersectional identities.
- Moderate: The proposed intervention would be integrated within communities and led by community health workers, which could increase the likelihood of sustainability.
- Moderate: This intervention could serve as a template for mental health interventions for other populations of African immigrant emerging adults

Weaknesses

- Moderate: It is not clear whether existing mental health interventions would be efficacious, and it is not clear which if any existing interventions the proposed intervention would be based on.

2. Investigator(s):

Strengths

- Major: The investigative team has expertise in adolescent and emerging adult health, community-based participatory intervention development and evaluation, and youth- focused mental health research.

Weaknesses

- None noted by reviewer.

3. Innovation:

Strengths

- Major: There are no mental health interventions for African immigrant youth in the US.

Weaknesses

- Minor: There are existing mental health interventions, but the team may not draw from these interventions in building the proposed one.

4. Approach:

Strengths

- Moderate: The proposed treatment fidelity monitoring and training procedures are strengths.
- Moderate: The proposed intervention uses a similar length, frequency, and format as other interventions developed for other immigrant populations.
- Moderate: The intervention will be provided in English and Amharic as appropriate.
- Moderate: The team successfully recruited survey participants within this population.
- Moderate: Appropriate acceptability, feasibility, and safety metrics.
- Moderate: Appropriate qualitative methodology, including the sample size rationale.
- Minor: Sex as a biological variable and other potential moderators will be considered.

Weaknesses

- Minor: It is not clear if weekly assessments are necessary and the incentives for these assessments may skew the attendance estimates (should the intervention be disseminated without weekly incentives).

5. Environment:

Strengths

- Major: The team has already engaged a youth advisory board and community-based organization, indicating community buy-in.
- Moderate: Appropriate academic resources for the investigators.

Weaknesses

- None noted by reviewer.

Study Timeline:

Strengths

- Appropriately detailed, sufficient time for activities

Weaknesses

- None noted by reviewer.

Protections for Human Subjects:

Acceptable Risks and/or Adequate Protections

- Balance of risk and benefit

Data and Safety Monitoring Plan (Applicable for Clinical Trials Only):

Acceptable

- Appropriate monitoring

Inclusion Plans:

- Sex/Gender: Distribution justified scientifically
- Race/Ethnicity: Distribution justified scientifically
- Inclusion/Exclusion Based on Age: Distribution justified scientifically
- No concerns

Vertebrate Animals:

Not Applicable (No Vertebrate Animals)

Biohazards:

Not Applicable (No Biohazards)

Resubmission:

- Responsive

Resource Sharing Plans:
Not Applicable (No Relevant Resources)

Budget and Period of Support:
Recommend as Requested

THE FOLLOWING SECTIONS WERE PREPARED BY THE SCIENTIFIC REVIEW OFFICER TO SUMMARIZE THE OUTCOME OF DISCUSSIONS OF THE REVIEW COMMITTEE, OR REVIEWERS' WRITTEN CRITIQUES, ON THE FOLLOWING ISSUES:

PROTECTION OF HUMAN SUBJECTS: ACCEPTABLE

INCLUSION OF WOMEN PLAN: ACCEPTABLE

INCLUSION OF MINORITIES PLAN: ACCEPTABLE

INCLUSION ACROSS THE LIFESPAN: ACCEPTABLE

COMMITTEE BUDGET RECOMMENDATIONS: The budget was recommended as requested.

Footnotes for 1R21MD019304-01A1; PI Name: Hussen, Sophia A.

+ Derived from the range of percentile values calculated for the study section that reviewed this application.

NIH has modified its policy regarding the receipt of resubmissions (amended applications).See Guide Notice NOT-OD-18-197 at https://grants.nih.gov/grants/guide/notice-files/NOT-OD-18-197.html. The impact/priority score is calculated after discussion of an application by averaging the overall scores (1-9) given by all voting reviewers on the committee and multiplying by 10. The criterion scores are submitted prior to the meeting by the individual reviewers assigned to an application, and are not discussed specifically at the review meeting or calculated into the overall impact score. Some applications also receive a percentile ranking. For details on the review process, see http://grants.nih.gov/grants/peer_review_process.htm#scoring.

MEETING ROSTER

Health Promotion in Communities Study Section
Social and Community Influences on Health Integrated Review Group
CENTER FOR SCIENTIFIC REVIEW
HPC
06/10/2024 - 06/11/2024

Notice of NIH Policy to All Applicants: Meeting rosters are provided for information purposes only. Applicant investigators and institutional officials must not communicate directly with study section members about an application before or after the review. Failure to observe this policy will create a serious breach of integrity in the peer review process, and may lead to actions outlined in NOT-OD-22-044 at https://grants.nih.gov/grants/guide/notice-files/NOT-OD-22-044.html, including removal of the application from immediate review.

CHAIRPERSON(S)

KEITA, AKILAH J, PHD
PROFESSOR AND CHAIR
DEPARTMENT OF SOCIAL, BEHAVIORAL, AND
POPULATION SCIENCES
SCHOOL OF PUBLIC HEALTH AND TROPICAL MEDICINE
TULANE UNIVERSITY
NEW ORLEANS, LA 70112

MEMBERS

ALLICOCK, MARLYN A, MPH, PHD *
ASSOCIATE PROFESSOR
HEALTH PROMOTION & BEHAVIORAL SCIENCES
UTHEALTH SCHOOL OF PUBLIC
HEALTH
DALLAS, TX 75207

AMUTAH-ONUKAGHA, NDIDIAMAKA, MPH, PHD *
ASSOCIATE PROFESSOR AND JULIA A. OKORO PROFESSOR
OF BLACK MATERNAL HEALTH
DEPARTMENT OF PUBLIC HEALTH AND COMMUNITY
MEDICINE
SCHOOL OF MEDICINE
TUFTS UNIVERSITY
BOSTON, MA 02111

CHEN, SENLIN, PHD *
PROFESSOR
SCHOOL OF KINESIOLOGY
LOUISIANA STATE UNIVERSITY
BATON ROUGE, LA 70803

CHEN-SANKEY, JULIA CEN, PHD *
ASSISTANT PROFESSOR
DEPARTMENT OF HEALTH BEHAVIOR, SOCIETY AND POLICY
RUTGERS SCHOOL OF PUBLIC HEALTH
PISCATAWAY, NJ 08854

DAVIS, RACHEL E, MPH, PHD
ASSOCIATE PROFESSOR
DEPARTMENT OF HEALTH PROMOTION,
EDUCATION, AND BEHAVIOR
ARNOLD SCHOOL OF PUBLIC HEALTH
UNIVERSITY OF SOUTH CAROLINA
COLUMBIA, SC 29208

EVANS, W DOUGLAS, PHD *
PROFESSOR
DEPARTMENT OF PREVENTION AND COMMUNITY HEALTH
AND GLOBAL HEALTH
MILKEN INSTITUTE SCHOOL OF PUBLIC HEALTH
THE GEORGE WASHINGTON UNIVERSITY
WASHINGTON, DC 20052

HALLOWAY, SHANNON, PHD
ASSOCIATE PROFESSOR
DEPARTMENT OF BIOBEHAVIORAL NURSING SCIENCE
COLLEGE OF NURSING
UNIVERSITY OF ILLINOIS, CHICAGO
CHICAGO, IL 60612

ISLAM, NADIA S, PHD
PROFESSOR
DEPARTMENT OF POPULATION HEALTH
NEW YORK UNIVERSITY SCHOOL OF MEDICINE
NEW YORK, NY 10016

KARR, CATHERINE J, MD, PHD
PROFESSOR
DEPARTMENTS OF PEDIATRICS AND
ENVIRONMENTAL AND OCCUPATIONAL HEALTH SCIENCES
UNIVERSITY OF WASHINGTON
SEATTLE, WA 98105

KRUKOWSKI, REBECCA A, PHD
PROFESSOR
DEPARTMENT OF PUBLIC HEALTH SCIENCES
SCHOOL OF MEDICINE
UNIVERSITY OF VIRGINIA
CHARLOTTESVILLE, VA 22908

LECHUGA, JULIA, PHD
ASSOCIATE PROFESSOR
DEPARTMENT OF PSYCHOLOGY
THE GRADUATE CENTER
HUNTER COLLEGE
THE CITY UNIVERSITY OF NEW YORK
NEW YORK, NY 10016

LEE, SUNMIN, MPH, SCD
PROFESSOR
DEPARTMENT OF MEDICINE
SCHOOL OF MEDICINE
UNIVERSITY OF CALIFORNIA, IRVINE
IRVINE, CA 92697

LYONS, ELIZABETH J, MPH, PHD
ASSOCIATE PROFESSOR AND CHAIR
DEPARTMENT OF NUTRITION SCIENCES AND
HEALTH BEHAVIOR
SCHOOL OF HEALTH PROFESSIONS
THE UNIVERSITY OF TEXAS MEDICAL BRANCH
GALVESTON, TX 77550

MAHONEY, JANE E, MD *
PROFESSOR
DEPARTMENT OF MEDICINE
SCHOOL OF MEDICINE AND PUBLIC HEALTH
UNIVERSITY OF WISCONSIN
MADISON, WI 53726

MAIR, CHRISTINA F, PHD
ASSOCIATE PROFESSOR
DEPARTMENT OF BEHAVIORAL AND
COMMUNITY HEALTH SCIENCES
SCHOOL OF PUBLIC HEALTH
UNIVERSITY OF PITTSBURGH
PITTSBURGH, PA 15261

MONTGOMERY, BROOKE, MPH, PHD *
ASSOCIATE PROFESSOR
DEPARTMENT OF HEALTH BEHAVIOR AND HEALTH
EDUCATION
UNIVERSITY OF ARKANSAS FOR MEDICAL SCIENCES
LITTLE ROCK, AR 72212

NELSON, TOBEN F, SCD
PROFESSOR
DIVISION OF EPIDEMIOLOGY
AND COMMUNITY HEALTH
SCHOOL OF PUBLIC HEALTH
UNIVERSITY OF MINNESOTA
MINNEAPOLIS, MN 55454

NESHTERUK, CODY, PHD *
ASSISTANT PROFESSOR
DEPARTMENT OF POPULATION HEALTH SCIENCES
DUKE UNIVERSITY SCHOOL OF MEDICINE
DURHMAN, NC 27701

OKAMOTO, SCOTT K, PHD *
PROFESSOR / RESEARCHER
POPULATION SCIENCES IN THE PACIFIC PROGRAM
UNIVERSITY OF HAWAI'I CANCER CENTER
HONOLULU, HI 96813

PATEL, MINAL R, PHD
PROFESSOR
DEPARTMENT OF HEALTH BEHAVIOR AND HEALTH
EDUCATION
SCHOOL OF PUBLIC HEALTH
UNIVERSITY OF MICHIGAN
ANN ARBOR, MI 48109

PFAMMATTER, ANGELA FIDLER, PHD *
ASSOCIATE PROFESSOR
DEPARTMENT OF PUBLIC HEALTH
THE UNIVERSITY OF TENNESSEE
KNOXVILLE, TN 37996

PICKETT, ANDREW C, PHD *
ASSISTANT PROFESSOR
DEPARTMENT OF HEALTH AND WELLNESS DESIGN
SCHOOL OF PUBLIC HEALTH- BLOOMINGTON
INDIANA UNIVERSITY
BLOOMINGTON, IN 47405

RALSTON, PENNY A, PHD
PROFESSOR
PROFESSOR & DIRECTOR, CENTER ON BETTER HEALTH
& LIFE FOR UNDERSERVED POPULATIONS
DEAN EMERITUS, COLLEGE OF HEALTH & HUMAN
SCIENCES
FLORIDA STATE UNIVERSITY
TALLAHASSEE, FL 32306

RISENDAL, BETSY C, PHD
PROFESSOR
DEPARTMENT OF COMMUNITY AND BEHAVIORAL HEALTH
COLORADO SCHOOL OF PUBLIC HEALTH
UNIVERSITY OF COLORADO
AURORA, CO 80045

ROSAS, LISA G, PHD
ASSISTANT PROFESSOR
DEPARTMENT OF EPIDEMIOLOGY AND POPULATION
HEALTH
DEPARTMENT OF MEDICINE
STANFORD UNIVERSITY SCHOOL OF MEDICINE
PALO ALTO, CA 94305

WANG, RUI, PHD
PROFESSOR
DEPARTMENT OF POPULATION MEDICINE
HARVARD PILGRIM HEALTH CARE INSTITUTE
HARVARD MEDICAL SCHOOL
BOSTON, MA 02215

WILLIAMS, LOVORIA B, PHD
PROFESSOR
COLLEGE OF NURSING
UNIVERSITY OF KENTUCKY
LEXINGTON, KY 40536

WINTERS-STONE, KERRI M, PHD
PROFESSOR
DIVISION OF ONCOLOGICAL SCIENCES
KNIGHT CANCER INSTITUTE
OREGON HEALTH AND SCIENCE UNIVERSITY
PORTLAND, OR 97239

SCIENTIFIC REVIEW OFFICER

DAGADU, HELENA ERYAM, MPH, PHD
SCIENTIFIC REVIEW OFFICER
CENTER FOR SCIENTIFIC REVIEW
NATIONAL INSTITUTES OF HEALTH
BETHESDA, MD 20892

* Temporary Member. For grant applications, temporary members may participate in the entire meeting or may review only selected applications as needed.

Consultants are required to absent themselves from the room during the review of any application if their presence would constitute or appear to constitute a conflict of interest.
